# Mild to moderate ulcerative colitis: Call me by my name

**DOI:** 10.1002/ueg2.12299

**Published:** 2022-08-28

**Authors:** Iago Rodríguez‐Lago, Manuel Barreiro‐de Acosta

**Affiliations:** ^1^ Gastroenterology Department Hospital Universitario de Galdakao Biocruces Bizkaia Health Research Institute Galdakao Spain; ^2^ School of Medicine University of Deusto Bilbao Spain; ^3^ Gastroenterology Department Hospital Clínico Universitario de Santiago Santiago de Compostela Spain

We are witnessing a progressive increase in the number of persons living with inflammatory bowel disease (IBD), including both Crohn's disease and ulcerative colitis (UC). Around 0.8% of the European population live with this disease, and this proportion is gradually rising every year.^1^ Thus, there is an urgent need to establish measures that will ensure quality of care and equality to all these patients in the near future.^2^ While the number of patients (unfortunately) increases, the treatment options have also grown in recent years. Those patients with difficult‐to‐treat disease have now more alternatives, but their disease often leads to more healthcare assistance—and consequently higher costs—so they are the main target of most clinical trials developing new compounds. Nonetheless, the European Epi‐IBD cohort showed that 68% of patients were classified as mild‐moderate during the first year, and only 5% fulfilled criteria for severe disease.^3^ These figures evolved optimistically over time, as up to 70% of patients were on clinical remission while one third were described as having mild to moderate disease after 4–5 years. Therefore, our daily practice will involve mainly the management of these patients, whereas there is no clearly standardized definition for this specific subgroup.

With this background, Caron and colleagues have nicely approached this issue through a systematic review,^4^ where they have evaluated the definitions of mild‐moderate UC in the clinical trials involving mesalazine or budesonide over the last 2 decades. Their effort is highly appreciated, as the current classification would embrace patients ranging from mild disease to those with higher inflammatory burden, and thus requiring steroids in some cases. The results from these authors confirm the tremendous heterogeneity defining this group of patients, with up to 6 scores used for this purpose with variable definitions and cut‐offs within each of them. The authors found that the most frequently used was the Ulcerative Colitis Disease Activity index (UCDAI), but it was applied in less than half of trials (41%). We are probably moving towards an standardized definition based on Mayo Clinic Score, as ongoing trials are more often using this measure^5^—also in clinical practice—, but all this evidence emphasizes that scientific societies and regulatory authorities need to work together here. This would be relevant from many points of view. Firstly, an homogeneous definition will help us compare results from epidemiological studies assessing disease severity along the disease course, which is critical in this type of long‐standing progressive diseases with a relapsing‐remitting course. Secondly, the design of registrational clinical trials have changed considerably and this can potentially influence results, interpretation, and comparability by itself^6^; so enrolling criteria in these studies should be as reliable and consistent as possible, hence reducing certain bias and improving external validity. Furthermore, subjects with moderate disease activity can fulfill inclusion criteria for trials looking for milder disease activity but also in those focused on more severe flares, and this can lead to certain bias in their results.

Nevertheless, the most recent clinical trials assessing drug efficacy in UC have already incorporated new items among their outcomes, both isolated or in combination.^7^ For instance, certain biomarkers (e.g., fecal calprotectin) are now considered formal targets of medical treatment, along with endoscopic healing, quality of life, and disability. Meanwhile, the evaluation of histological disease activity has been reported with the newly developed compounds, but it is still not considered a formal target in the STRIDE‐II recommendations.^8^ The perspective on these targets has changed since the original version published back in 2015, so we can expect further changes in the upcoming years as evidence about the impact of each outcome is updated. Thus, the definition of disease activity should be in line with the outcomes and our overall understanding of the disease. Furthermore, we will need also to re‐consider how we can assess the full picture of the disease, because those factors involving mental well‐being should also be included in the equation, as independent elements influencing the perception of the severity and quality of life.^9^, ^10^

We would like to commend the authors and highlight their great work, as they also have evaluated predictors of response to mesalazine or budesonide in subjects with mild to moderate disease. Apart from those aspects associated with a higher inflammatory burden, shorter disease duration remains as a relevant factor. Taken together, all these results suggest that earlier intervention in milder patients is highly relevant, which is essential when considering chronic diseases like UC. The future is expected to incorporate these advances and to bring us new tools and approaches to the treatment of UC, that will ultimately lead to improving outcomes in the long‐term.

## CONFLICTS OF INTEREST

There are no conflicts of interest related to this manuscript.

## FUNDING INFORMATION

Gobierno Vasco‐Eusko Jaurlaritza, Grant/Award Number: 2020222004.
